# High Proteoglycan Decorin Levels Are Associated With Acute Coronary Syndrome and Provoke an Imbalanced Inflammatory Response

**DOI:** 10.3389/fphys.2021.746377

**Published:** 2021-09-21

**Authors:** Lingfang Zhuang, Yulong Ge, Xiao Zong, Qian Yang, Ruiyan Zhang, Qin Fan, Rong Tao

**Affiliations:** ^1^Department of Cardiovascular Medicine, Ruijin Hospital, Shanghai Jiao Tong University School of Medicine, Shanghai, China; ^2^Institute of Cardiovascular Diseases, Shanghai Jiao Tong University School of Medicine, Shanghai, China; ^3^Department of Cardiology, School of Medicine, Shanghai General Hospital, Shanghai Jiao Tong University, Shanghai, China

**Keywords:** acute coronary syndrome, decorin, biomarker, ischemic heart disease, inflammatory response, white blood cells, high-sensitivity C-reactive protein

## Abstract

**Background and Aims:** Acute coronary syndrome (ACS) has become one of the most common causes of disability. It is thus important to identify ACS early in the disease course of patients using novel biomarkers for prompt management. Decorin (DCN) was well-acknowledged for its effect on collagen fibrillogenesis and maintaining tissue integrity. Additionally, DCN could release as secreted proteoglycan under pathological conditions. Hence, we aimed to determine the relationship between serum DCN concentration and ACS.

**Methods:** A total of 388 patients who underwent coronary angiography (CAG) in the cardiovascular center of Ruijin Hospital between June 2016 and December 2017 were enrolled in this study. Blood samples were drawn during CAG surgery to determine the serum DCN level of patients with ACS (*n* = 210) and control subjects (*n* = 178) using enzyme-linked immunosorbent assay.

**Results:** We found that the serum DCN levels of ACS patients were elevated compared with those of the control subjects (13.59 ± 0.50 vs. 13.17 ± 0.38, respectively, *p* < 0.001). Furthermore, the serum DCN level, after being adjusted with other cardiovascular factors, was independently associated with ACS. Moreover, an increased serum DCN level was positively correlated with the number of white blood cells and the level of high-sensitivity C-reactive protein (*R* = 0.3 and 0.11, respectively). Mechanistically, DCN might have elicited an imbalanced inflammatory response during cardiac ischemia by suppressing the expression of anti-inflammatory genes.

**Conclusion:** Serum DCN is a novel biomarker of ACS and contributes to the increased inflammatory response in ischemic heart disease.

## Introduction

Acute coronary syndrome (ACS) is caused by the sudden rupture or erosion of a vulnerable atherosclerotic coronary plaque, which then results in narrowed or occluded coronary vessels and reduced blood flow to the heart (Anderson and Morrow, 2017). According to the Global Burden of Disease Study 2019, ACS has the highest disability-adjusted life-years since 1990 (GBD 2019 Diseases and Injuries Collaborators, 2020). Thus, there is an urgent need to diagnose ACS in its early stages using novel biomarkers for prompt coronary revascularization.

Decorin (DCN) is a chondroitin sulfate proteoglycan that interacts with other proteoglycans. It constitutes the extracellular matrix (ECM); thus, it affects the structure and stiffness of the ECM (Fransson et al., 2000; Vu et al., 2018). Although DCN is a scaffolding protein that supports the integrity of the heart, DCN can be released in its soluble form under pathological conditions which suggests that liberated soluble DCN in the blood circulation is a danger sign (Frey et al., 2013). Previous studies have found that serum DCN level is associated with preterm premature rupture of fetal membranes (Underhill et al., 2019), acute ischemic stroke (Xu et al., 2012), sepsis (Merline et al., 2011), and coronary artery disease (Nazemi et al., 2018), suggesting serum DCN level may be used as a biomarker in the diagnosis of various diseases. In addition, Barallobre-Barreiro et al. (2016) have shown that an increase in the full-length of DCN or its N-terminal domain is associated with atrial fibrillation. Notably, in a preclinical model, researchers have found that DCN participates in the remodeling process of myocardial infarction by inducing abnormal collagen fibrotic evolution, which further compromises scar tissue (Weis et al., 2005), or by enhancing cardiomyocyte survival after stimulated ischemia/reperfusion injury (Gaspar et al., 2020). These findings suggest the association between DCN and myocardial infarction. Moreover, Jahanyar et al. (2007) demonstrate that both the mRNA and protein expression of cardiac DCN are upregulated in patients with heart failure after mechanical circulatory. In these patients, DCN potentially interferes with the p-SAMD2 pathway. Medeiros et al. (2002) showed that upregulated DCN expression in spontaneously hypertensive heart failure (SHHF) rat hearts and in congestive heart failure hearts. These findings suggest the crucial role of DCN in heart failure. Although pre-clinical or clinical studies have linked DCN with myocardial infarction or cardiac dysfunction, there are no data on the diagnostic value of serum DCN in ACS patients. Therefore, in this study, we aimed to compare the serum DCN level between ACS patients and control subjects and to elucidate the inflammatory response of DCN during myocardial infarction.

## Materials and Methods

### Patient Characteristics

A total of 388 patients who underwent coronary angiography (CAG) between June 2016 and December 2017 at the cardiovascular center of Ruijin Hospital were enrolled in this study. The exclusion criteria of the study were as follows: (1) patients with less than 1 year of life expectancy; (2) those with malignancy; (3) those with acute infection; and (4) those with severe liver or kidney dysfunction. The participants’ clinical symptoms, CAG results, electrocardiogram (ECG) findings, and serum troponin I levels were obtained and were used to classify the participants into two groups: the group consisting of patients with ACS and that consisting of control subjects. Those with intact coronary arteries or with < 50% coronary stenosis were assigned to the control group. Contrastingly, the criteria for being classified into the ACS group were as follows: (1) acute chest pain; (2) ECG results showing ST-segment elevation, T-wave inversion, or pathological Q wave; (3) increased serum troponin I level (> 0.5 ng/mL); and (4) > 50% stenosis of an epicardial coronary artery seen in CAG. ACS patients were further worked-up to identify whether they have ST-elevation myocardial infarction (STEMI), non-ST-elevation myocardial infarction (NSTEMI), or unstable angina (UA). All patients in the ACS group underwent percutaneous coronary intervention (PCI) during catheterization operations. This study was carried out in accordance with the ethical guidelines of the 1975 Declaration of Helsinki and the study protocol has been priorly approved by the Ruijin Hospital Ethics Committee on research on humans (The ethics committee reference number: 2016-019). All patients provided written informed consent.

### Decorin Measurement and Data Collection

Blood samples were collected from the patients during CAG. After the samples were stored at room temperature for 2 h, the serum was centrifuged at 3,000 rpm for 20 min, while the serum was stored at −40°C before the assay was conducted. The DCN concentration was quantitatively determined using an *in vitro* enzyme-linked immunosorbent assay (ELISA) kit from RayBiotech (ELH-Decorin). The blood samples were diluted at 1:100, which was in accordance with the manufacturer’s instructions. The ELISA assay was conducted by a technician who was blinded to the clinical data of the involved patients by using the numbered ID method.

The levels of hemoglobin, hemoglobin A1c, white blood cell (WBC), high-density lipoprotein (HDL), low-density lipoprotein (LDL), and high-sensitivity C-reactive protein (hsCRP) levels were measured from the peripheral venous blood samples via routine laboratory methods. Troponin I (cTnI) levels were measured within 6 h of hospital admission and serially thereafter. The cTnI levels before CAG or PCI operations were included in the present study.

### Mononuclear Isolation and Treatment

Blood samples from ACS patients were collected and stored into EDTA-containing tubes. Mononuclear cells (MNCs) from the blood samples were isolated via density-gradient centrifugation according to the manufacturer’s instructions (Lymphoprep^TM^, Stemcell Technologies, #07801). Briefly, blood samples were diluted with Dulbecco’s Modified Eagle Medium (DMEM) at a ratio of 1:1. The diluted samples were then layered on top of the Lymphoprep buffer by centrifugation at 800 g for 30 min (ACC: DEC = 9: 1). The MNCs were subsequently isolated from the interphase and washed twice with phosphate buffered saline (PBS) twice. Cells were seeded in six-well plates and cultured with Roswell Park Memorial Institute Medium 1640 supplemented with 10% fetal bovine serum, 100 U/mL penicillin, 100 μg/mL streptomycin, and 10 ng/mL recombinant human macrophage colony-stimulating factor (PeproTech, Cranbury, NJ, United States) for 24 h. Recombinant human DCN protein (R&D Systems) was incubated with MNCs (20 ng/mL or 60 ng/mL) for 24 h. Total RNA was then extracted using the TRIzol reagent (Thermo Fisher Scientific) as previously described (Zhuang et al., 2019).

Primers for quantitative real-time polymerase chain reaction (RT-qPCR) used in this study are listed below.

*ARG1* (Forward: 5′-ACTTAAAGAACAAGAGTGTGATGTG-3′; Reverse: 5′-GCATCCACCCAGATGACTCC-3′),

*SPP1* (Forward: 5′-AGCAGAATCTCCTAGCCCCA-3′; Reverse: 5′-ACGGCTGTCCCAATCAGAAG-3′),

*CCL24* (Forward: 5′-GCTGTCACCCTGTTACCTCC-3′; Reverse: 5′-GAGCCCGTAGGGATGATGTG-3′),

*MRC1* (Forward: 5′-AATGGCATGAAGCGGAGACA-3′; Reverse: 5′-ATTCCAGAGAAGCTTGGCCC-3′),

*IL-6* (Forward: 5′-GTCCAGTTGCCTTCTCCCTGG-3′; Reverse: 5′-CCCATGCTACATTTGCCGAAG-3′),

*INOS* (Forward: 5′-GCCAGGCCACCTCTATGTTT-3′; Reverse: 5′-GAGGCTCCGATCAATCCAGG-3′),

*MARCO* (Forward: 5′-CTTCTCCCTAGCTGTGGTGG-3′; Reverse: 5′-GCATCTCCTTTCATGCCCCT-3′),

*TNF* (Forward: 5′-CTGGGCAGGTCTACTTTGGG-3′; Reverse: 5′-CTGGAGGCCCCAGTTTGAAT-3′),

*GAPDH* (Forward: 5′-AATGGGCAGCCGTTAGGAAA-3′; Reverse: 5′-GCGCCCAATACGACCAAATC-3′).

### Statistical Analyses

Continuous data are hereby presented as mean ± standard deviation (SD), as indicated in the legends, while categorical variables are expressed as frequency in percentage. To obtain a normal distribution, we transformed the DCN level by a logarithm of two for further analysis (Supplementary Figures 2A,B). After the normality test, the Student’s *t*-test was applied when data were normally distributed; otherwise, the Mann-Whitney *U* test was used to compare the statistical differences in continuous variables. On the other hand, the chi-square test was used for categorical variables. The correlations between DCN and WBC, DCN and hsCRP were determined using the Spearman’s rank correlation coefficient in Rstudio. Univariable and multivariable logistic regression analyses were further conducted to explore the link between DCN and ACS with or without adjusting for sex, age, smoking, hypertension, diabetes, systolic blood pressure, WBC, HbA1c, total cholesterol, HDL, LDL, creatinine, hsCRP, and ln-transformed cTnI, as indicated in the legend. To demonstrate the diagnostic value of DCN, the receiver operating characteristic curve (ROC), area under ROC (AUROC), and clinical risk factors with or without DCN were calculated. The ROC plot included the true-positive fraction (sensitivity) and the false-positive fraction (1-specificity) was generated using the R package pROC. All tests were two-sided, and a *P*-value < 0.05 was considered statistically significant. All statistical analyses were performed in the Statistical Package for the Social Sciences (SPSS) version 23 (International Business Machines Corporation, Armonk, NY, United States) or R version 3.6.1.

## Results

### The Source and Distribution of Decorin and the Patients’ Baseline Characteristics

To determine which cell type expressed DCN in the heart, we investigated the level of DCN in our integrated single-cell RNA-sequencing data, which were consisted of fibroblasts, macrophages, neutrophils, endothelial cells, and pericytes (Zhuang et al., 2020). We found that DCN was virtually expressed in fibroblasts (Supplementary Figure 1A). Furthermore, the immunofluorescence co-staining of DCN and vimentin, the fibroblast marker, was consistent with that of the single-cell RNA-sequencing data and showed co-localization of DCN with vimentin (Supplementary Figure 1B).

**FIGURE 1:**
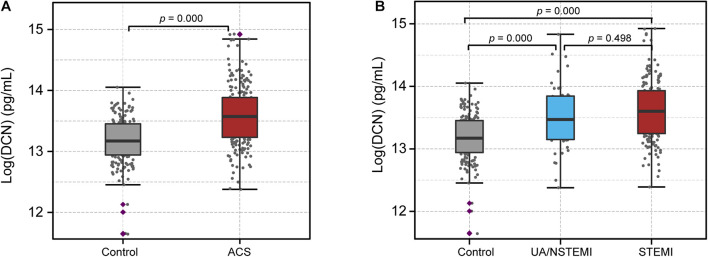
Serum level of DCN increased in ACS patients. **(A)** The serum level of DCN in patients with acute coronary syndrome (ACS) compared with the control subjects. **(B)** The serum level of DCN in the control, UA/NSTEMI, and STEMI subjects.

The serum levels of DCN were then measured from 388 patients and were transformed logarithmically for further analysis (Supplementary Figures 2A,B). The clinical characteristics of the patients are listed in Table 1. All patients were divided into two groups: the control group (*n* = 178) and the ACS group (*n* = 210) according to their CAG, ECG, and troponin I results. Age, sex, body mass index, history of hypercholesterolemia, history of myocardial infarction, diastolic blood pressure, heart rate, hemoglobin, and triglycerides were similar between the two groups. However, there were statistical differences in terms of smoking history, systolic blood pressure, left ventricular ejection fraction (LVEF), WBC, neutrophil percentage (NE%), hemoglobin A1c (HbA1c), total cholesterol, HDL, LDL, creatinine, cTnI, hsCRP, and presence of hypertension and diabetes between ACS and the control patients. Notably, the serum DCN levels in the ACS group were significantly increased compared with those of the control group (13.59 ± 0.50 vs. 13.17 ± 0.38; *p* = 0.000, Figure 1A). Furthermore, we compared the DCN levels of NSTEMI/UA and STEMI patients with those of the control subjects. Although higher DCN levels were found under ischemic conditions, the serum DCN remained comparable between the STEMI and NSTEMI groups (Figure 1B).

**TABLE 1:** Baseline characteristics of Acute coronary syndrome (ACS) and control subjects.

	Control *n* = 178	ACS *n* = 210	*p*-value
Age (y), mean (SD)	65.0(9.74)	67.2(12.11)	0.053
Male, n (%)	140(78.7)	178(84.5)	0.119
BMI (kg/m^2^), mean (SD)	24.8(3.29)	25.0(3.83)	0.768
Smoking, n (%)	57(32.0)	91(43.3)	0.022
**Medical history**			
Hypertension, n (%)	93(52.2)	135(64.3)	0.016
Diabetes, n (%)	18(10.1)	45(21.4)	0.003
Hypercholesterolemia, n (%)	10(5.60)	17(8.10)	0.339
Previous MI/PCI, n (%)	5(2.80)	15(7.10)	0.054
**Physiological Parameters, mean (SD)**			
Systolic BP (mmHg)	132.8(17.1)	127.1(20.1)	0.003
Diastolic BP (mmHg)	76.9(11.6)	75.9(13.0)	0.409
Heart rate (BPM)	77.5(11.8)	80.1(15.3)	0.165
LVEF (%)	65.8(5.95)	58.6(7.89)	0.000
**Laboratory examinations, mean (SD)**			
Hemoglobin (g/L)	140(14.4)	140.5(18.0)	0.610
WBC (x10^9/L)	6.28(1.96)	9.10(3.14)	0.000
NE (%)	56.9(9.98)	67.9(15.2)	0.000
HbA1c (%)	5.94(0.86)	6.32(1.37)	0.016
Triglycerides (mmol/L)	1.63(1.93)	1.67(0.97)	0.059
Total cholesterol (mmol/L)	4.16(1.00)	4.46(1.58)	0.011
HDL (mmol/L)	1.24(0.80)	1.02(0.23)	0.000
LDL (mmol/L)	2.55(1.59)	2.75(0.97)	0.002
Creatinine (μmol/L)	77.6(16.77)	85.3(27.77)	0.008
cTnI (ng/mL)	0.01(0.032)	39.75(89.3)	0.000
hsCRP (mg/L)	3.04(8.31)	17.3(27.55)	0.000
DCN (pg/mL)	13.17(0.38)	13.59(0.50)	0.000
**Medication, n (%)**			
ACEI/ARB	51(28.7)	154(73.3)	0.000
β-Blocker	100(56.2)	175(83.3)	0.000
CCB	73(41.0)	23(11.0)	0.000
Statin	112(62.9)	201(95.7)	0.000
anti-platelet drugs	140(78.5)	210(100)	0.000

*Data for DCN were transformed in logarithm of two. Continuous data were presented as mean (Standard Deviation, SD); Category data were presented as number (percentage, %).*

*p-value was obtained using Chi-squared Test (for category data), or Student-t test, Mann-Whitney U test (for continuous data) as appropriate.*

*ACS, acute coronary syndrome; BMI, body mass index; BP, blood pressure; WBC, white blood cell; HbA1c, Hemoglobin A1c; HDL, high density lipoprotein; LDL, low density lipoprotein; hsCRP, high sensitivity C reactive protein; CCB, calcium channel blocker.*

**FIGURE 2:**
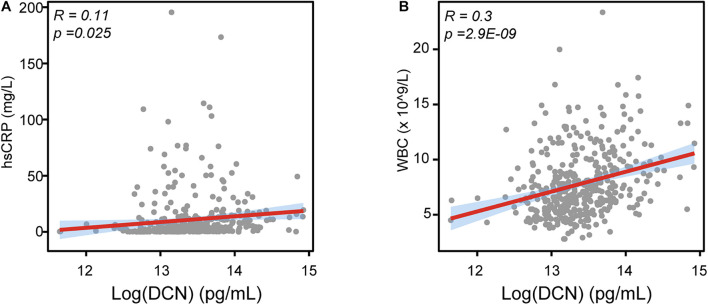
The relationship between DCN with LVEF, WBC, and hsCRP. **(A)** The correlation between serum DCN with the serum level of hsCRP in enrolled patients. **(B)** The correlation between serum DCN with the number of white blood cells (WBC) in enrolled patients. R referred to correlation coefficient.

We then determined the correlation between clinical risk factors and serum DCN levels. Although no statistical difference was found between DCN concentration with gender, hypercholesterolemia history, smoking, serum DCN levels were significantly elevated in patients with hypertension and diabetes history (Table 2). Notably, a higher DCN level was significantly correlated with elevated hsCRP (Figure 2A, *R* = 0.11, *p* = 0.025) and WBC (Figure 2B, *R* = 0.3, *p* = 2.9E-09) levels.

**TABLE 2:** Association of cardiovascular risk factors with DCN concentrations.

	Log (DCN)	*p*-value
**Gender**		
Male (*n* = 318)	13.386 ± 0.509	*p* = 0.542
Female (*n* = 70)	13.435 ± 0.421	
**Hypertension**
Yes (*n* = 228)	13.455 ± 0.512	*p* = 0.002
No (*n* = 160)	13.309 ± 0.457	
**Diabetes**
Yes (*n* = 63)	13.549 ± 0.491	*p* = 0.020
No (*n* = 325)	13.365 ± 0.490	
**Hypercholesterolemia**
Yes (*n* = 27)	13.388 ± 0.482	*p* = 0.771
No (*n* = 361)	13.396 ± 0.496	
**Smoking**
Yes (*n* = 148)	13.411 ± 0.528	*p* = 0.475
No (*n* = 240)	13.386 ± 0.473	
**hsCRP (*R* = 0.11, *p* = 0.025)**
≤ 2.5 (*n* = 202)	13.245 ± 0.422	*p* = 0.000
> 2.5 (*n* = 186)	13.558 ± 0.516	
**WBC (*R* = 0.3, *p* = 2.9E-09)**
Q1: ≤ 6 (*n* = 117)	13.213 ± 0.415	*p*^*a*^ = 0.000
Q2: 6–10 (*n* = 191)	13.421 ± 0.494	*p*^*b*^ = 0.037
Q3: ≥ 10 (*n* = 79)	13.604 ± 0.516	*p*^*c*^ = 0.000

*Data were presented as Mean ± SD.*

*Differences between two groups were compared with Mann-Whitney U test. P^*a*^ compared the level of DCN in Q1 group with Q2 group using Mann-Whitney U test, P^*b*^ compared Q2 with Q3, and P^*c*^ compared Q1 with Q3.*

*The correlation analysis between continuous hsCRP or WBC level with DCN concentration was compared using Pearson correlation coefficient in R.*

### The Diagnostic Value of Decorin

**FIGURE 3:**
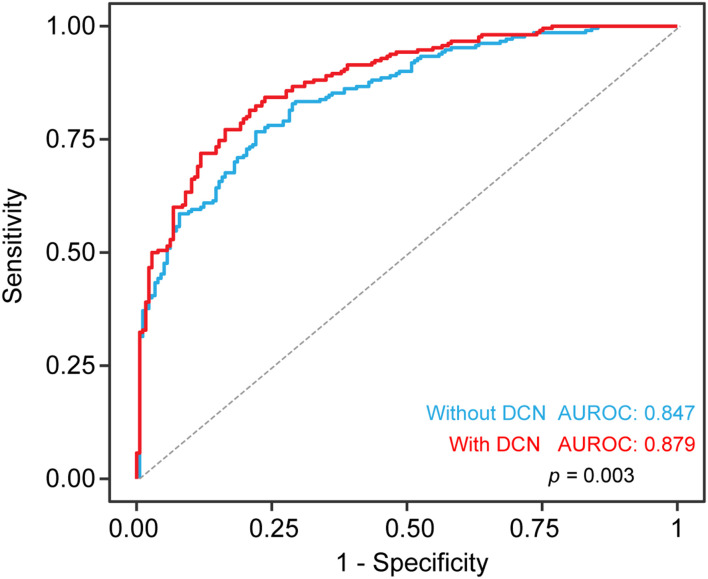
Diagnostic accuracy for ACS with serum DCN. The receiver operating characteristic curve (ROC) including clinical risk factor with or without serum DCN for the diagnostic value of ACS in all patients. Risk factors includes age, sex, smoking, hypertension, diabetes, systolic BP, WBC, HbA1c, total cholesterol, HDL, LDL, creatinine. Sensitivity (y axis) and 1-specificity was plotted as indicated.

Since we showed that serum DCN levels were increased in ACS patients, we aimed to determine the diagnostic value of DCN especially in patients suspected with ACS. Using univariable and multivariable logistic regression analyses, we found that log-transformed DCN was positively correlated with the odds of ACS [OR: 9.093, 95% confidence interval (CI) = 5.105–16.196, *p* = 0.000]. This relationship persisted even after the adjustment for age, sex, smoking history, hypertension, diabetes, systolic blood pressure, WBC, HbA1c, total cholesterol, HDL, LDL, creatinine, hsCRP, and ln-transformed cTnI, as shown in Table 3 (OR: 9.393, 95% CI = 1.770–49.849, *p* = 0.009). The diagnostic accuracy of DCN was confirmed using the ROC curve. Adding DCN to the multivariate logistic model statistically increased the diagnostic accuracy of DCN in discriminating patients with ACS from control patients (Figure 3, *p* = 0.003) with specificity at 83.6% and sensitivity at 77.1% and the optimal threshold value of DCN was 13.65 (pg/mL, log-transformed). These findings suggest that serum DCN may act as a novel biomarker that discriminates patients with ACS from those who do not.

**TABLE 3:** Univariable and multivariable logistic analysis on the association of DCN and other clinical parameters with ACS.

Parameters	Univariate			Multivariate		
	OR	95% CI	*p*-value	OR	95% CI	*p*-Value
Age	1.018	0.999−1.036	0.059			
Sex	0.662	0.394−1.114	0.120			
Smoking	1.623	1.070−2.462	0.023			
Hypertension	1.645	1.094−2.473	0.017			
Diabetes	2.424	1.346−4.366	0.003			
Systolic BP	0.984	0.973−0.994	0.003			
WBC	1.623	1.444−1.824	0.000			
HbA1c	1.378	1.121−1.693	0.002			
Cholesterol	1.292	1.068−1.564	0.008			
LDL	1.171	0.956−1.434	0.127			
Creatinine	1.016	1.006−1.027	0.002			
hsCRP	1.120	1.077−1.164	0.000			
HDL	0.059	0.024−0.148	0.000	0.000	0.000−0.024	0.000
cTnI	6.697	4.119−10.890	0.000	13.743	5.790−32.622	0.000
DCN	9.093	5.105−16.196	0.000	9.393	1.770−49.849	0.009

*Binary logistic analysis revealed the association of DCN and other clinical parameters with ACS by using univariate logistics analysis.*

*Multivariate logistic analysis revealed the association of DCN with ACS after adjusting for age, sex, smoking, hypertension, diabetes, systolic BP, WBC, HbA1c, total cholesterol, HDL, LDL, creatinine, hsCRP, and ln-transformed cTnI.*

*OR, odds ratio; 95% CI, 95% confidence interval.*

### Decorin Aggravated Inflammatory Response in Acute Coronary Syndrome Patients

**FIGURE 4:**
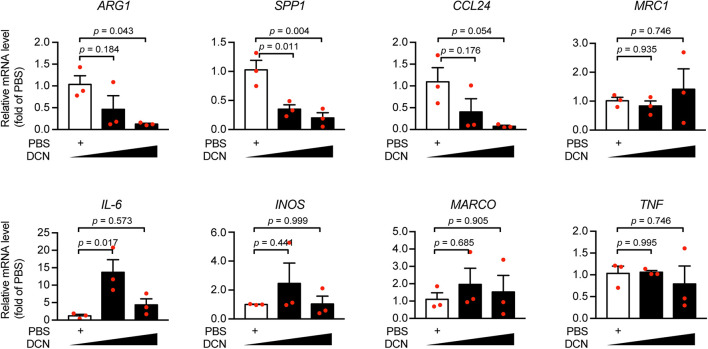
DCN elicited pro-inflammatory responses under ischemic conditions. Human mononuclear cells (MNC) were treated with recombinant human DCN protein (20 ng/mL, or 60 ng/mL) or PBS and the mRNA expression of *ARG1, SPP1, CCL24, MRC1, IL-6, INOS, MARCO, TNF* was determined by quantitative real-time polymerase chain reaction (RT-qPCR).

Furthermore, we sought to interrogate the effects of DCN-induced inflammatory response in myocardial infarction. Since the association of DCN with collagen fibrillogenesis and cardiac fibrosis was already extensively studied (Bianco et al., 1990; Reed and Iozzo, 2002; Weis et al., 2005), we did not explore the role of DCN in fibrosis in the present study. We have described previously that serum DCN was positively correlated with WBC numbers (Figure 2B), therefore we investigated at the cellular level the effect of DCN on the inflammatory phenotypes by isolating peripheral blood MNCs from ACS patients and by treating them with different concentrations of DCN. We found that DCN treatment dramatically downregulated the mRNA expression of anti-inflammatory genes, such as *ARG1*, *SPP1*, and *CCL24*, in a concentration-dependent manner (Figure 4). Although *IL-6* was sharply increased after DCN treatment, the other pro-inflammatory genes remained similar between the group treated with DCN and that treated with PBS (Figure 4). These findings suggest that DCN can induce inflammation under ischemic cardiac conditions by suppressing the pro-repair phenotype of MNCs.

## Discussion

In the present study, we found that DCN, which was exclusively expressed in cardiac fibroblasts, was remarkably elevated in the serum after myocardial infarction. The results showed that DCN could independently distinguish patients with ACS from those who without. Furthermore, treatment with exogenous human DCN recombinant protein resulted in the upregulation of the mRNA expression of pro-inflammatory genes such as *IL-6* and the downregulation of anti-inflammatory genes such as *ARG1, SPP1*, and *CCL24* in MNCs of ACS patients.

DCN belongs to the family of small leucine-rich proteoglycan family (Iozzo, 1999). It is primarily named after the eponym of decoration on the basis of its capacity to bind with collagen I at regular intervals and to regulate the synthesis of collagen fibrils under physiological and pathological conditions (Bianco et al., 1990; Reed and Iozzo, 2002; Merline et al., 2009). The anti-fibrotic role of DCN is evident in the avid interaction among DCN, TGF-β (Schonherr et al., 1998; Kolb et al., 2001), and connective tissue growth factor (Vial et al., 2011) which further sequester the bioactivity of TGF-β and connective tissue growth factor. By acting as an endogenous inhibitor of TGF-β, DCN attenuates ECM deposition and fibrosis in myocardial infarction (Weis et al., 2005) and glomerulonephritis models (Border et al., 1992). Moreover, researches in the last few decades have explored the biological function of DCN as an injury marker in various diseases. It is known that DCN is sequestered by the ECM under physiological conditions; however, under pathological conditions, it is released in its soluble form from the ECM via proteolysis or via *de novo* synthesis by activated cells. Thus, DCN can act as a danger signal and a biomarker of tissue stress and injury (Frey et al., 2013). In this study, we found that serum DCN levels were significantly elevated in ACS patients compared with those in control subjects and showed reliable value for the diagnosis of ACS. In fact, the previous studies have linked DCN with cardiac diseases, indicating DCN is elevated in patients with atrial fibrillation (Barallobre-Barreiro et al., 2016) and in those with heart failure (Jahanyar et al., 2007). These studies highlight the indispensable role of DCN in the pathophysiology of cardiac diseases. Consistent with the results of our study, Neufeld et al. have demonstrated that DCN can interact with LDL in the disease-prone vascular wall and can induce LDL uptake and retention into the subendothelial matrix in a concentration-dependent manner. These findings emphasize the indispensable role of DCN in the development of atherosclerosis (Neufeld et al., 2014). These results are confirmed by ours, which show that DCN is positively correlated with the onset of myocardial infarction.

Mechanistically, we found that elevated DCN was positively correlated with the number of white blood cells, indicating a relationship between DCN and inflammation. Using human MNCs from the peripheral blood of ACS patients, we further showed that DCN treatment elicited an imbalanced anti- and pro-inflammatory gene profile, which might account for the detrimental role of DCN in the progression of ACS. DCN treatment resulted in the upregulation of *IL-6* and downregulation of *ARG1*, *SPP1*, and *CCL24*. In fact, this is not the first study that links DCN with inflammation. Merline et al. has demonstrated that DCN can act as an endogenous ligand of Toll-like receptors 2 (TLR2) and TLR4 in the sepsis. By binding to TLR2/TLR4, DCN triggers inflammatory responses via P38, mitogen-activated protein kinases (MAPK), and nuclear factor-κB pathways to stimulate the expression and release of programmed cell death 4, TNF-α, and IL-12, while inhibiting the level of IL-10, a cytokine with anti-inflammatory properties. Furthermore, deletion of DCN mitigates the pro-inflammatory cytokine profile in sepsis and sterile inflammatory diseases (Merline et al., 2011). In addition, biglycan, another proteoglycan related to DCN, shows pro-inflammatory effects similar to those of DCN through the TLR2/TLR4-MyD88 pathway to boost the levels of TNF-α and macrophage inflammatory protein and prompt the infiltration of MNCs in the lungs in lipopolysaccharide- and zymosan-induced sepsis (Schaefer et al., 2005). These data are consistent with those of ours and reveal the pro-inflammatory role of DCN in myocardial infarction.

However, this study has some limitations, (1) Although we described the emerging diagnostic role of DCN with ACS, we did not investigate the prognostic role of DCN after myocardial infarction. Thus, further studies are needed to delineate the risk stratification role of DCN in ACS patients. (2) We did not delineate the underlying pathways induced by DCN that lead to an inflammatory response. Further studies should focus on the downstream pathways of DCN-induced inflammatory responses and their association with fibrosis during cardiac remodeling.

This study demonstrated the diagnostic value of serum DCN in the setting of myocardial infarction. We showed that elevated DCN was associated with ACS. Furthermore, we found that DCN had a pro-inflammatory function, thereby indicating that serum DCN could function as a novel biomarker and therapeutic modality in ACS.

## Data Availability Statement

The original contributions presented in the study are included in the article/Supplementary Material, further inquiries can be directed to the corresponding author/s.

## Ethics Statement

The studies involving human participants were reviewed and approved by the Ruijin Hospital Ethics Committee on research on humans. The patients/participants provided their written informed consent to participate in this study.

## Author Contributions

LZ: methodology, investigation, formal analysis, and writing—original draft. YG: methodology, formal analysis, and writing—review and editing. XZ: methodology and formal analysis. QY: investigation and formal analysis. RZ: investigation and formal analysis. QF: formal analysis and writing—review and editing. RT: conceptualization, investigation, and writing—review and editing. All authors contributed to the article and approved the submitted version.

## Conflict of Interest

The authors declare that the research was conducted in the absence of any commercial or financial relationships that could be construed as a potential conflict of interest.

## Publisher’s Note

All claims expressed in this article are solely those of the authors and do not necessarily represent those of their affiliated organizations, or those of the publisher, the editors and the reviewers. Any product that may be evaluated in this article, or claim that may be made by its manufacturer, is not guaranteed or endorsed by the publisher.
