# Supplementary material for: High Proteoglycan Decorin Levels Are Associated With Acute Coronary Syndrome and Provoke an Imbalanced Inflammatory Response
Source: Front Physiol. 2021 Sep 21;12:746377. doi: 10.3389/fphys.2021.746377 (PMC8490816; doi:10.3389/fphys.2021.746377)
Supplement: Supplementary file 1 [file Data_Sheet_1.docx]

Supplementary Material

# Supplementary Figures

**
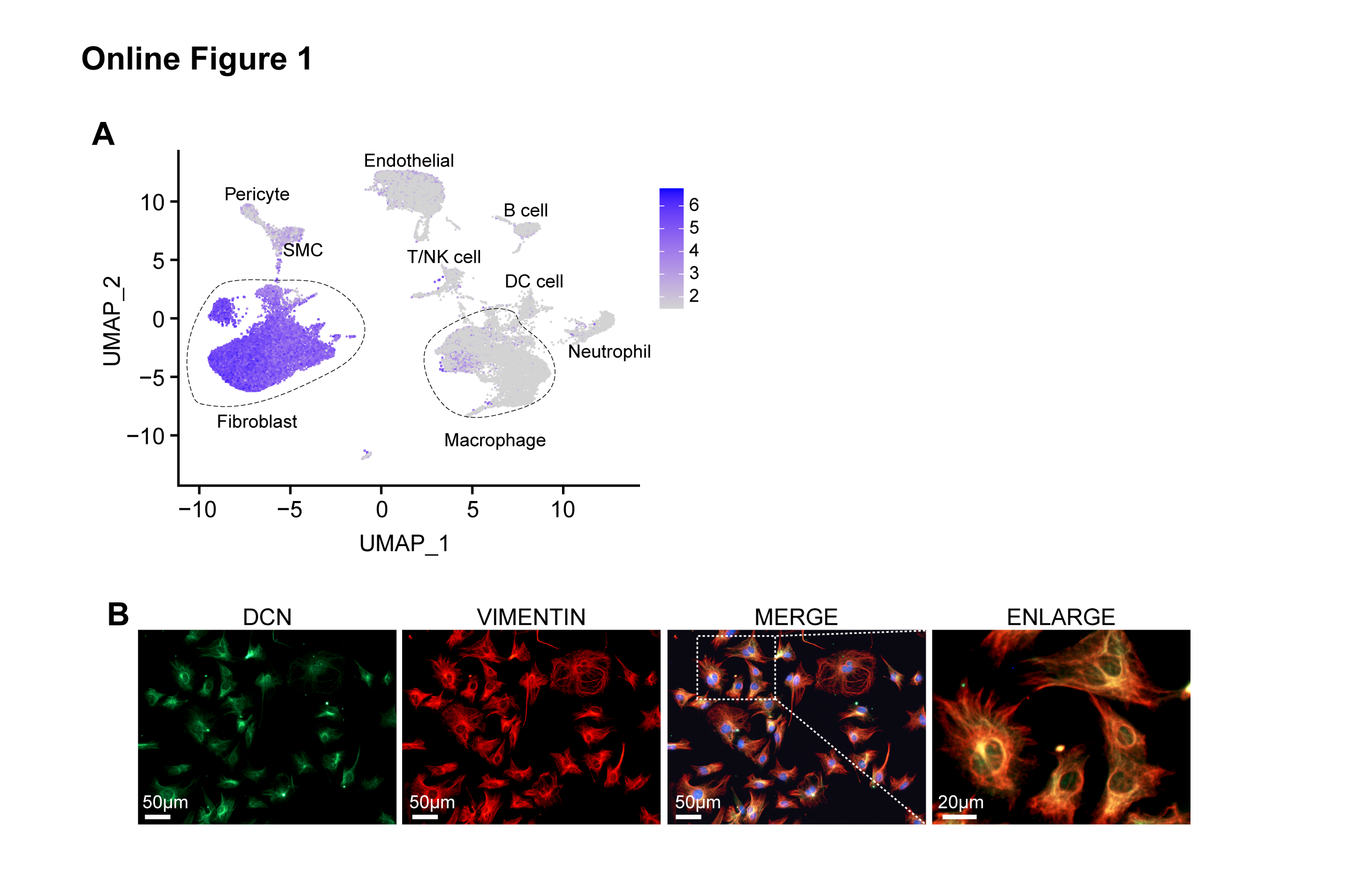
Online Figure 1. The expression pattern of DCN.** (A) The expression of DCN was interrogated in our single-cells RNA-sequencing data. (B) Co-staining of DCN (green) and VIMENTIN (red) showed expression of DCN in fibroblasts.

**
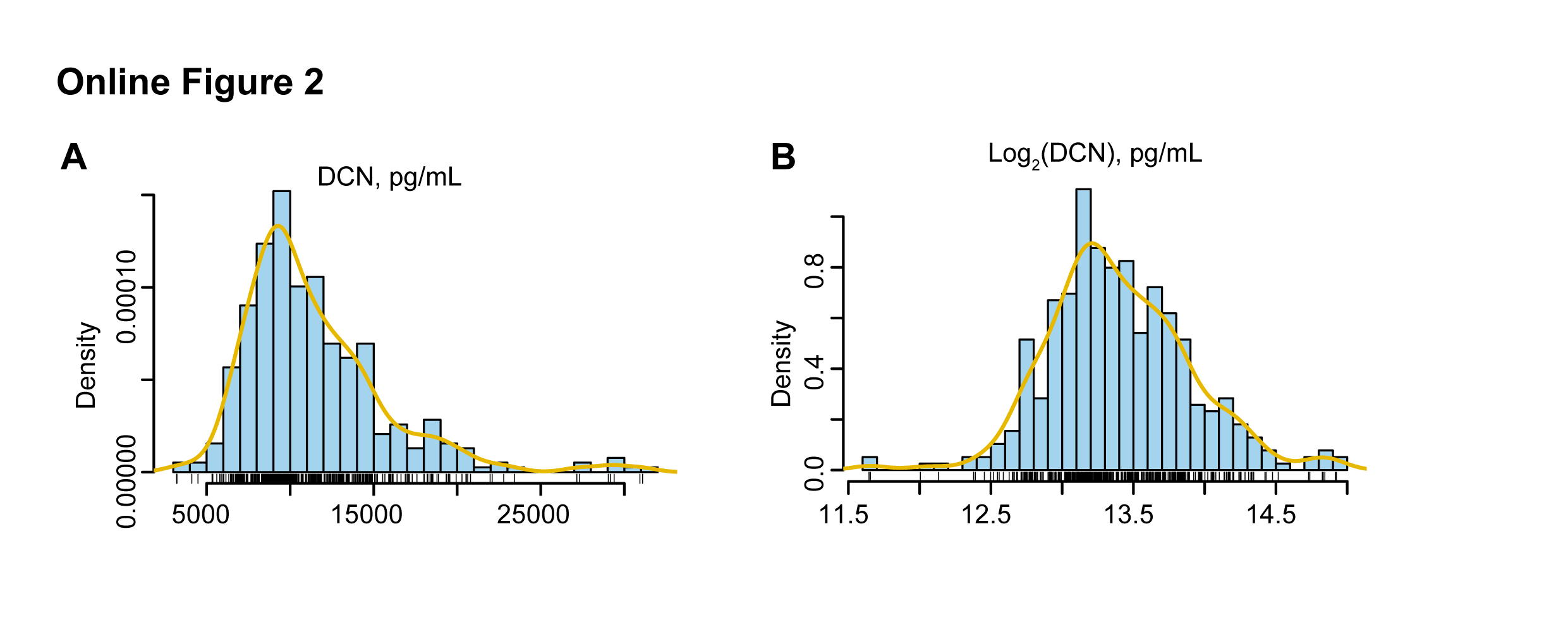
**

**Online Figure 2. Transformation of DCN with logarithm for normality.** (A) The histogram showed the distribution of DCN in all patients. (B) The level of DCN was transformed with logarithm of 2, and the level of DCN was showed as histogram plot.
